# Supplementary material for: Drug repurposing for aging research using model organisms
Source: Aging Cell. 2017 Jun 16;16(5):1006–15. doi: 10.1111/acel.12626 (PMC5595691; doi:10.1111/acel.12626)
Supplement: Supplementary file 7 — Data S1 Zip‐Archive of all report cards. [file ACEL-16-1006-s007.zip › RC_BA1.pdf]

BA1

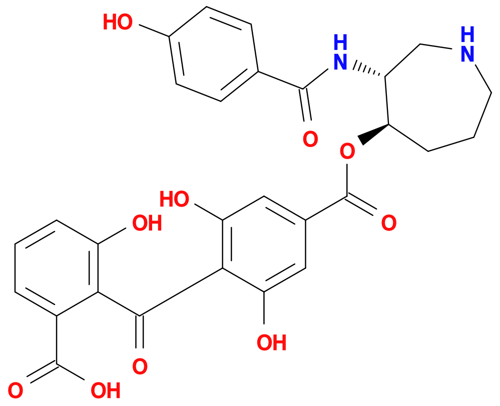

Database identifiers

ChEMBLCompound	CHEMBL60254
eMolecules	8741645

Ranking

	Rank	Score
Drosophila	339/697	0.441
C. elegans	504/591	0.066

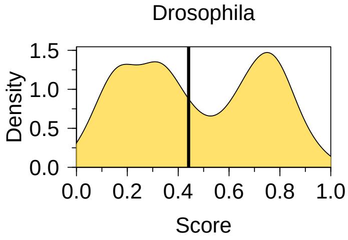

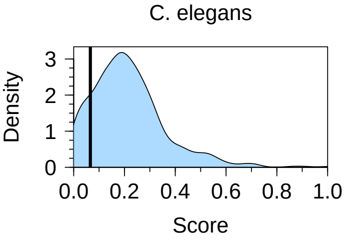

	Ageing implication	Domain conservation	Binding site conservation	Binding affinity	Bioavailability	Lipinski	Promiscuity	Purchasability	Drug approval	**Total**
Drosophila	0.624	0.969	1.0	0.903	(0.9)	-0.15	-0.0	0.1	0.0	0.441
C. elegans	0.624	0.97	1.0	0.903	0.212	-0.15	-0.0	0.1	0.0	0.066

Names

- 2-2,6-dihydroxy-4-(3R,4R)-3-(4-hydroxybenzoyl)aminoazepan-4-yloxycarbonylbenzoyl-3-hydroxybenzoic acid
- 2-2,6-dihydroxy-4-(3R,4R)-3-(4-hydroxyphenyl)-oxomethylamino-4-azepanyloxy-oxomethylphenyl-oxomethyl-3-hydroxybenzoic acid
- 2-2,6-dihydroxy-4-(3R,4R)-3-(4-hydroxyphenyl)carbonylaminoazepan-4-yloxycarbonyl-phenylcarbonyl-3-hydroxy-benzoic acid
- Balanol
- MEGxm0$_0$00504

Roles

ChEBI entry None has no roles

Status

Approved drug (according to ChEMBL)	No
Number of Rule of 5 violations	3
Binding affinity to original target in log units (RF-Score prediction)	7.23
Burns *C. elegans* bioavailability prediction	-5.36

Compound Target Characteristics

Beta-adrenergic receptor kinase 1

Best gene implication in ageing for this target family came from gene F1LMA4 via mapping the annotation from RGD 2062 annotated in RGD 2014-03-11. Annotation GO 7568 (aging) was Inferred from Expression Pattern

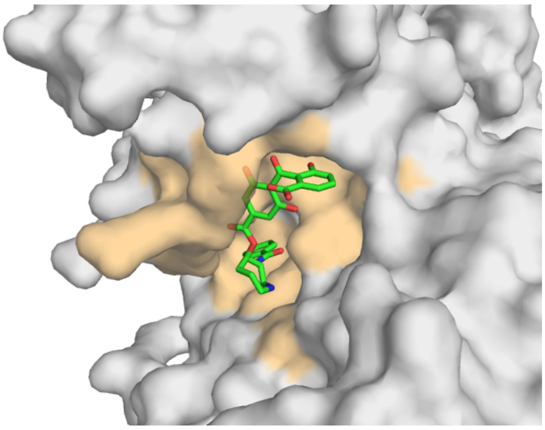

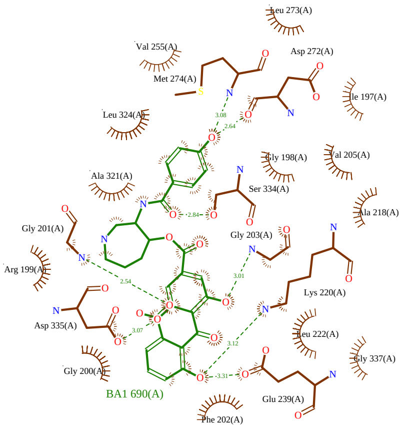

protein	amino acids contacts (binding site)
PDB:3krw:chainA:P25098	I G R G G F G E V A K L E V D L M A L S D G
PDB:3krx:chainA:P25098	I G R G G F G E V A K L E V D L M A L S D G
tr:E9PRV7:E9PRV7_HUMAN	I G R G G F G E V A K L E V D L M A L S D G
sp:P25098:ARBK1_HUMAN	I G R G G F G E V A K L E V D L M A L S D G
tr:F1LMA4:F1LMA4_RAT	I G R G G F G E V A K L E V D L M A L S D G
tr:E9PW16:E9PW16_MOUSE	I G R G G F G E V A K L E V D L M A L S D G
tr:Q7TS64:Q7TS64_MOUSE	I G R G G F G E V A K L E V D L M A L S D G
tr:Q3U1V3:Q3U1V3_MOUSE	I G R G G F G E V A K L E V D L M A L S D G
sp:Q99MK8:ARBK1_MOUSE	I G R G G F G E V A K L E V D L M A L S D G
tr:E1JGX2:E1JGX2_DROME	I G R G G F G E V A K L E V D L M A L S D G
sp:P32865:GPRK1_DROME	I G R G G F G E V A K L E V D L M A L S D G
sp:Q09639:GRK2_CAEEL	I G R G G F G E V A K L E V D L M A L S D G

	whole protein		domain-based		contact-based	
protein	ident	simil	ident	simil	ident	simil
PDB:3krw:chainA:P25098	1.0	1.0	1.0	1.0	1.0	1.0
PDB:3krx:chainA:P25098	1.0	1.0	1.0	1.0	1.0	1.0
tr:E9PRV7:E9PRV7_HUMAN	0.54	0.56	0.65	0.66	1.0	1.0
sp:P25098:ARBK1_HUMAN	1.0	1.0	1.0	1.0	1.0	1.0
tr:F1LMA4:F1LMA4_RAT	0.94	0.94	1.0	1.0	1.0	1.0
tr:E9PW16:E9PW16_MOUSE	0.53	0.56	0.65	0.66	1.0	1.0
tr:Q7TS64:Q7TS64_MOUSE	0.93	0.94	1.0	1.0	1.0	1.0
tr:Q3U1V3:Q3U1V3_MOUSE	0.99	1.0	1.0	1.0	1.0	1.0
sp:Q99MK8:ARBK1_MOUSE	0.99	1.0	1.0	1.0	1.0	1.0
tr:E1JGX2:E1JGX2_DROME	0.64	0.87	0.84	0.94	1.0	1.0
sp:P32865:GPRK1_DROME	0.64	0.87	0.84	0.94	1.0	1.0
sp:Q09639:GRK2_CAEEL	0.62	0.85	0.84	0.95	1.0	1.0

Gprk1 (FBgn0260798) associated phenotypes

heat sensitive, neurophysiology defective

(Information from FlyBase)

Gprk1 (UniProt:P32865) annotation
Function: Specifically phosphorylates the activated forms of G protein-coupled receptors.
(Information from UniProt)

grk-2 (WBGene00001709) associated phenotypes
aqueous negative chemotaxis variant, bitter tastant response variant, copper response variant, diacetyl chemotaxis defective, isoamyl alcohol chemotaxis defective, octanol chemotaxis defective, osmotic avoidance defective, pyrazine chemotaxis defective, quinine chemotaxis defective
(Information from WormBase)

grk-2 (UniProt:Q09639) annotation
Function: Specifically phosphorylates the activated forms of G protein-coupled receptors.
(Information from UniProt)
